# Distributed training of CosPlace for large-scale visual place recognition

**DOI:** 10.3389/frobt.2024.1386464

**Published:** 2024-05-20

**Authors:** Riccardo Zaccone, Gabriele Berton, Carlo Masone

**Affiliations:** Visual And Multimodal Applied Learning Laboratory (VANDAL Lab), Dipartimento di Automatica e Informatica (DAUIN), Politecnico di Torino, Turin, Italy

**Keywords:** visual place recognition, visual geolocalization, distributed learning, image retrieval, deep learning

## Abstract

Visual place recognition (VPR) is a popular computer vision task aimed at recognizing the geographic location of a visual query, usually within a tolerance of a few meters. Modern approaches address VPR from an image retrieval standpoint using a kNN on top of embeddings extracted by a deep neural network from both the query and images in a database. Although most of these approaches rely on contrastive learning, which limits their ability to be trained on large-scale datasets (due to mining), the recently reported CosPlace proposes an alternative training paradigm using a classification task as the proxy. This has been shown to be effective in expanding the potential of VPR models to learn from large-scale and fine-grained datasets. In this work, we experimentally analyze CosPlace from a continual learning perspective and show that its sequential training procedure leads to suboptimal results. As a solution, we propose a different formulation that not only solves the pitfalls of the original training strategy effectively but also enables faster and more efficient distributed training. Finally, we discuss the open challenges in further speeding up large-scale image retrieval for VPR.

## 1 Introduction

Visual place recognition (VPR) (Masone and Caputo, 2021) is a popular computer vision task that aims to recognize the geographic location of a visual query and usually has an accepted tolerance of a few meters. VPR tasks are commonly approached as image-retrieval problems, in which a never-before-seen query image is matched to a database of geotagged images; the most similar images in the database are then used to infer the coordinates of the query.

The typical pipeline for VPR involves a neural network to extract embeddings from both the query and each image in the database. These embeddings are then compared using a k-nearest neighbor (kNN) algorithm to retrieve the most similar results from the database and their corresponding geotags. For the kNN step to be effective, it is crucial that the embedding space learned by the neural network be sufficiently discriminative for places; this is commonly achieved by training the models with contrastive learning approaches using a triplet loss (Arandjelović et al., 2018) or other similar losses and leveraging the geotags of the database images as a form of weak supervision to mine negative and positive examples (Arandjelović et al., 2018). However, the execution time required for the mining operation scales linearly with the size of the database (Berton et al., 2022b), thus becoming a bottleneck that impedes training on massive datasets. A naive mitigation strategy here would be to mine the positive/negative examples within a subset of the data (Warburg et al., 2020), but this ultimately hampers the ability to learn more discriminative and generalizable representations.

To solve this problem at its root, Berton et al. (2022a) recently proposed a paradigm shift in the training procedure for VPR. Their solution called CosPlace is specifically designed for large-scale and fine-grained VPR, and it adopts a classification task as the proxy for training the model without mining. To enable this classification proxy, CosPlace introduces a partitioning strategy that divides the continuous label space of the training images (GPS and compass annotations) into a finite set of disjoint groups (CosPlace groups), each containing a number of classes. This partition is intended to guarantee that images from different classes (i.e., representative of different places) within the same group have no visual overlap. Thereafter, CosPlace is trained sequentially on a single group at a time to avoid ambiguities caused by partition-induced visual aliasing (Figure 2, left). Although CosPlace can be trained on a much larger number of images than reported in previous works and has achieved new state-of-the-art (SOTA) results, we hypothesize that the sequential training protocol is suboptimal because it optimizes an approximation of the intended minimization problem. This hypothesis stems from approaching the CosPlace training protocol from an incremental learning perspective. In fact, each CosPlace group may be regarded as a separate learning task that uses a shared feature extractor and a per-group classification head. During each epoch, the model is trained for a given number of optimization steps on a single group (task). However, there is no guarantee that switching to a new task during the next epoch will not harm the model performances for the older tasks. In this paper, we experimentally validate this hypothesis by showing that sequential training delays convergence and that there are eventually diminishing returns as the number of groups increases beyond a certain threshold.

**FIGURE 1:**
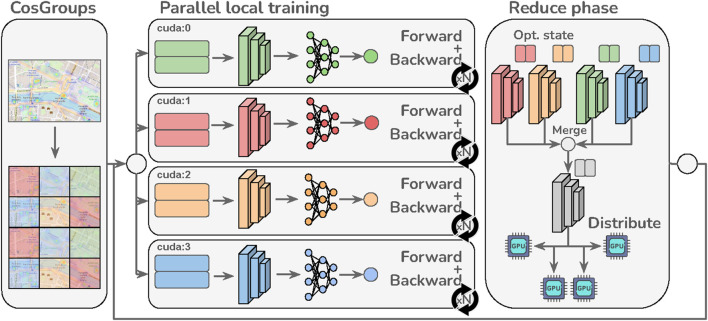
In the proposed D-CosPlace, each accelerator parallelly optimizes the model with respect to a different CosGroup for *J* steps before merging the model and optimizers’ states (backbone only). This process is repeated until convergence.

**FIGURE 2:**
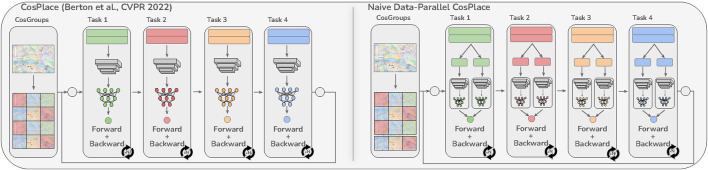
Comparison of CosPlace (Berton et al., 2022a) with a naive data-parallel variant. Unlike both approaches, the model in the proposed solution is jointly optimized with respect to all the training CosGroups (Figure 1). Best viewed in color.

In light of this observation, we redefine the CosPlace training procedure so that the algorithm trains different groups parallelly (Figure 1). Note that this is different from applying a standard data parallel approach since this would only split the same batch of data corresponding to the same task among the available accelerators (Figure 2, right). The proposed solution not only solves the previous issue by implementing joint objective optimization over all the selected groups but also allows efficient training parallelization. Hence, we refer to this solution as distributed-CosPlace (D-CosPlace). The main contributions of this work are summarized as follows:

• We analyze CosPlace to unveil the pitfalls of the original sequential formulation and investigate possible mitigation strategies.
• We propose a new group-parallel training protocol called D-CosPlace, which not only addresses extant issues but also allows effective use of communication-efficient SOTA distributed algorithms. This improves the performance of the original CosPlace by a large margin on several VPR datasets within the same time budget.
• By further analyzing the training of the proposed distributed version of CosPlace, we outline the open challenges in speeding up training for large-scale VPR.

## 2 Related works

### 2.1 Large-scale visual place recognition

Modern VPR approaches extract compact image embeddings using a feature extractor backbone followed by a head that implements aggregation or pooling (Kim et al., 2017; Arandjelović et al., 2018; Ge et al., 2020; Ali-bey et al., 2023; Berton et al., 2023; Zhu et al., 2023). These usually employ contrastive learning, using the geotags of the training set as a type of weak supervision to mine negative examples. However, this mining operation is expensive and impractical for scaling to large datasets (Berton et al., 2022b). To mitigate this problem, Ali-bey et al. (2022) proposed the use of a curated training-only dataset in which the images are already split into predefined classes that are far apart from each other, thereby enabling the composition of training batches with images from the same place (positive examples) and from other places (negative examples) very efficiently. The method proposed by Leyva-Vallina et al. (2023) involves annotating the images with a graded similarity, thus enabling training with contrastive losses and full supervision while achieving improvements in terms of both data efficiency and final model quality. Instead of mitigating the cost of mining, Berton et al. (2022a) proposed an approach to remove it entirely through their CosPlace method. The idea of CosPlace is to first partition the training images into disjoint groups with one-hot labels and to then train sequentially on these groups with the CosFace loss (Wang et al., 2018) that was originally designed for large-scale face recognition. Although CosPlace achieves SOTA results on large-scale datasets and even in generalized scenarios, we show here that its sequential training procedure is suboptimal and hampers the convergence speed. In view of these findings, we introduce a parallel-training version of CosPlace that improves the convergence speed and produces new SOTA results on several benchmarks.

### 2.2 Distributed training

The growth of deep-learning methods and training datasets is driving research on distributed training solutions. Among these, data parallelism constitutes a popular family of methods (Lin et al., 2020) wherein different chunks of data are processed in parallel before combining the model updates either synchronously or asynchronously. In particular, to reduce the communication overhead of data movement between the accelerators, local optimization methods are commonly used to allow multiple optimization steps on disjoint sets of data before merging the updates (Stich, 2019; Yu et al., 2019; Wang et al., 2020). In this work, we redefine CosPlace’s training procedure by introducing the parallel training of groups and leveraging local methods to speed up convergence.

## 3 Analysis of CosPlace

In this section, we analyze the CosPlace training algorithm and highlight the drawbacks of its sequential protocol.

### 3.1 Notation

The first step in CosPlace’s training protocol involves creating a set of discrete labels from the continuous space of the Universal Transverse Mercator (UTM) coordinates of the area of interest (Berton et al., 2022a). Formally, we define the training distribution D≔X×C , where X is the space of possible images and C is the space of UTM coordinates (east, north, heading). We also define a new distribution $\hat{D}≔X\times Y$ , where Y is the label space induced by partitioning C . Formally, a UTM point c∈C is discretized to a label $y=\left\{\left\lfloor \frac{east}{M}\right\rfloor ,\left\lfloor \frac{north}{M}\right\rfloor ,\left\lfloor \frac{heading}{\alpha }\right\rfloor \right\}$, where *M* and *α* describe the extent of a region covered by any class in meters and degrees, respectively. The set of such classes is then split into groups called CosGroups by fixing the minimum spatial separation between two classes of the same group in terms of both translation and orientation. Formally, a CosPlace group is defined as the set of classes such that

$$\begin{array}{ll} G_{u,v,w} & ≔\\ & \left\{y\in Y:\;\left\lfloor \frac{east}{M}\right\rfloor \;\mathrm{mod}\;N=u,\;\left\lfloor \frac{north}{M}\right\rfloor \;\mathrm{mod}\;N=v,\;\left\lfloor \frac{heading}{\alpha }\right\rfloor \;\mathrm{mod}\;L=w\right\}, \end{array}$$ (1)

where *N* and *L* are hyperparameters for the fixed minimum spatial and angular separations between classes belonging to the same CosGroup. We denote the set of such groups as G , i.e., $G=\left\{G_{u,v,w}\right\}\;\forall u,v,w\in N$ . Given multiple CosGroups (defined by Eq. 1), it is possible to derive multiple training distributions $\hat{D_{i}}≔X\times G_{i}\subset \hat{D}$ , where each distribution maps the sample image to a one-hot label within the *i*th CosGroup. The CosGroups partition is reflected in the model and is composed of two components: a feature extractor $B\left(\cdot \right):X\to R^{D}$ parameterized by weights *θ* ^*b*^ and multiple classifiers $F_{i}\left(\cdot \right):R^{D}\to {\left[0,1\right]}^{\left|G_{i}\right|}$ that are each associated with a different CosGroup parameterized by the weights $\theta_{i}^{f}$ .

### 3.2 CosPlace objective function

The goal of CosPlace is to learn a feature extractor *B*(⋅) that maps the original distribution X in an embedding space such that the distances between the locations depicted in the images are reflected well. Therefore, CosPlace aims to optimize the following problem:

$${\theta^{b}}^{*}=\text{arg}\min_{\theta^{b}}\overset{\left|G\right|}{\underset{i=1}{\sum }}E_{\left(x,y\right)∼\hat{D_{i}}}\left[L_{\mathrm{lmcl}}\left(F_{i}◦B,\left(x,y\right)\right)\right].$$ (2)

In practice, the training procedure should minimize the large margin cosine loss (LMCL) (Wang et al., 2018) of the entire model $\theta ≔\left\{\theta^{b},\cup_{i}\theta_{i}^{f}\right\}$ with respect to the label distribution(s) induced by discretization of the GPS coordinates into classes and by the grouping of these classes. The parameters $\theta_{i}^{f}$ of the classifiers are used only to train the feature extractor *θ* ^*b*^ and discarded after training. The final performances of *B*(⋅) are assessed using the kNN algorithm as the proxy with respect to the original distribution D .

### 3.3 CosPlace training: a continual learning perspective

Although CosPlace aims to optimize Eq. 2, it is observed that the sequential optimization of *θ* ^*b*^ with respect to each CosGroup is just an approximation of this objective function. Formally, it implements

$$\begin{array}{ll} {\theta_{G_{i}}^{b}}^{*} & =\text{arg}\min_{\theta^{b}}E_{\left(x,y\right)∼\hat{D_{i}}}\left[L_{\mathrm{lmcl}}\left(F_{i}◦B,\left(x,y\right),\theta_{G_{i-1}}^{b^{\;\;\;*}}\right)\right]\;\forall i\in \left[1,|\bar{G}|\right]\\ {\theta_{G_{0}}^{b}}^{*} & =\theta_{0}^{b}\;\left(initial\;model\right), \end{array}$$ (3)

where $\bar{G}\subseteq G$ is a subset of all possible CosGroups selected *a priori* for training. Eq. 3 practically means that at each *iteration* *e*, the training procedure selects the *i*th CosGroup $G_{i}$ , with $i≔\left(e\;\mathrm{mod}\;|\bar{G}|\right)$ , and jointly optimizes the parameters *θ* ^*b*^ and $\theta_{i}^{f}$ for *s* optimization steps starting from the optimal model obtained from the previous CosGroup $G_{i-1}$ .

**FIGURE 3:**
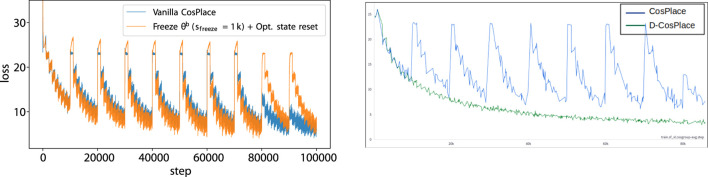
Training instabilities of CosPlace (left) and solution using D-CosPlace (right): changing classifiers (e.g., each *s* =10*k* steps) is followed by a spike in the training loss. Simple mitigation strategies, e.g., freezing *θ* ^*b*^ for a number of *s*_*freeze*_ steps to warmup the classifier and resetting the optimizers’ states, have limited efficacy and do not work in the long run. The proposed D-CosPlace is unaffected by this problem by design since all the classifiers are optimized jointly.

By expressing the CosPlace learning problem in this form, we can revisit it from a continual learning perspective. Accordingly, each distribution associated with a CosGroup can be considered as a task with a disjoint set of labels and dedicated parameters $\theta_{i}^{f}$ . Therefore, when CosPlace training iterates to a new CosGroup, it is akin to switching to a new task (Figure 2, left). This is different from solving the original problem in Eq. 2 because there is no guarantee that switching to the new task will not harm the model performances for the older tasks. In practice, the new model updates could be detrimental to the previous tasks, a phenomenon known as catastrophic forgetting (Goodfellow et al., 2014; Pfülb and Gepperth, 2019; Ramasesh et al., 2021). To verify if this phenomenon actually manifests during CosPlace training, we performed an experiment using its original implementation on the SF-XL dataset provided by Berton et al. (2022a). We plot the training loss for this experiment in Figure 3, from which it can be clearly seen that at each iteration, when switching to a new CosGroup, the loss function exhibits a steep increase and requires many steps to recover a loss value similar to the one before group change. This behavior is especially notable in the first few iterations, after which it disappears gradually as it is expected for the model to achieve convergence.

The reason why optimizing Eq. 3 still works remarkably well is that the CosPlace training protocol relies on the fact that each task will be revisited after some iterations. Therefore, the algorithm eventually converges to a solution that is also good for the joint objective function of Eq. 2. However, this is achieved at the cost of increased training time and is hardly scalable with respect to the number of trained groups $\left|\bar{G}\right|$ , as observed in the original work (Berton et al., 2022a). Together, these problems drastically limit the training time scalability of CosPlace, which is its main purpose.

### 3.4 Mitigation strategies

Given that the most severe jumps in the training loss in Figure 3 occur in the first few iterations, i.e., when the classifiers $\theta_{i}^{f}$ associated with each task have not yet been trained, one can consider some engineering solutions to solve this problem. A first modification would be to freeze the backbone model *θ* ^*b*^ for a number of steps *s*_*freeze*_ ≪ *s* whenever the task is changed. This prevents the weights $\theta_{i}^{f}$ from being uninitialized or too stale with respect to the backbone. Additionally, considering the amount of training that the model *θ* ^*b*^ has undergone since the last time task *i* was selected, it would also be beneficial to reset the optimizer state for model $\theta_{i}^{f}$ as it may be excessively biased. However, repeating the same experiment as before with these modifications shows that the effectiveness is limited (Figure 3, orange line). In particular, we observe that resetting the optimizer step is only beneficial during the first few iterations, which slightly speeds up convergence. However, we find that this strategy worsens the final model quality in the long run because maintaining the optimizer states is beneficial as the model finally approaches convergence. A similar observation also holds for freezing *θ* ^*b*^; it is initially useful, although a very large number of *s*_*freeze*_ steps are needed for a noticeable reduction in the training loss. In the long run, this becomes detrimental because these steps are wasted.

In conclusion, despite their simplicity, such simple mitigation strategies require careful engineering to determine *s*_*freeze*_ as well as decide when to use them, making them practically ineffective. Moreover, since these issues arise after performing a significant amount of training between two samplings of the same task *i*, these simple strategies cannot be scaled when the number of training CosGroups $\left|\bar{G}\right|$ increases.

## 4 Distributed CosPlace

The analysis presented in Section 3 reveals that the CosPlace training procedure does not correctly implement the objective function of Eq. 2. The problem here lies in the sequential protocol, which optimizes the model with respect to each CosGroup separately in a sequential manner. To recover the objective function of Eq. 2, we should calculate the gradients for all CosGroups in parallel, i.e., using the same model *θ* ^*b*^, before averaging them to update the model according to the optimizer policy. These gradients can be computed sequentially or in parallel to benefit from the multiple accelerators. This joint optimization procedure exactly recovers the original objective function of the vanilla CosPlace aimed at optimization Eq. 2: indeed, at each optimization step, the algorithm optimizes $E_{\left(x,y\right)∼\hat{D_{i}}}\left[L_{\mathrm{lmcl}}\left(F_{i}◦B,\left(x,y\right)\right)\right]$ jointly with respect to all CosGroups $G_{i}\in \bar{G}$ . Accordingly, the proposed formulation effectively addresses the problem outlined in Section 3.3 as shown in Figure 3 (right), in which the severe jumps during the sequential learning of CosGroup are solved completely.

This idea may seem to be similar to standard data parallelization, as implemented in most deep-learning frameworks. In fact, a common implementation would entail dividing the original batch of data into *k* smaller chunks, letting each accelerator compute gradients with respect to the same model on a chunk, merging these chunks, and updating the final model according to the optimizer policy (Figure 2, right). However, this approach does not address the problem arising from sequential training as noted previously because it would still be applied separately to each CosGroup. Instead, we need a data parallelization strategy that is aware of the divisions in the CosGroups where each one corresponds to a separate classifier and can jointly optimize the model with respect to all CosGroups. Moreover, since each CosGroup is a disjoint set of data by construction, it is possible to assign one or more CosGroups to each accelerator or compute node and train without the need of a distributed sampling strategy or centralized storage. This effectively reduces data movement related to the training samples because a CosGroup can be previously stored locally on its assigned compute node.

This group-parallel approach can be further improved using local optimization methods (Stich, 2019; Lin et al., 2020). The core idea here is to have a master send the current model *θ* ^*b*^ to all accelerators that parallelly optimize it for *J* (local) steps before returning the updates to the master. The master then averages the updates and applies them to the current model. This process is repeated for a given number of iterations until convergence. Intuitively, performing multiple local steps before averaging allows training speedup by reducing the communication rate between the accelerators. It is also important to note that pure local methods allow the use of any optimizer during local training, while the master always calculates the new model as an exact average of the local models after training. A more general approach is SlowMo (Wang et al., 2020) that further applies stochastic gradient descent (SGD) with momentum on the master by using the exact average of the trainers’ gradients as the pseudogradient. Trivially, setting the momentum term *β* = 0 in SlowMo corresponds to recovering the pure local method employed. By implementing multiple local steps, using local methods on CosGroup allows i) respecting the problem formulation in Eq. 2, ii) lowering the data movement related to training samples, and iii) achieving high communication efficiency during training. A scheme representing the parallel training procedure across different CosGroups using local methods is depicted in Figure 1, which we call as the D-CosPlace system.

## 5 Experiments

### 5.1 Implementation details

#### 5.1.1 Model and training datasets

For all the experiments, we used a backbone based on ResNet-18, followed by GeM pooling and a fully connected layer with output dimension *D* = 512, as in Berton et al. (2022a). As per the training dataset, we used SF-XL, a large-scale dataset created from Google StreetView imagery, and retained the best hyperparameters of the original CosPlace (*M* = 10 m, *α* = 30°, *N* = 5, and *L* = 2). Under this configuration, the total number of CosGroups is |G|=50 , and training is performed through experiments with $|\bar{G}|\in \left\{4,8,16\right\}$ , thereby demonstrating that the proposed approach can be scaled with increasing number of groups (and hence the dataset size).

#### 5.1.2 Training hyperparameters

For the classic CosPlace sequential training, *s* = 10*k* iterations for a given CosGroup before moving on to the next. As optimizers, Adam and Local-Adam are used for the distributed version, with learning rates of *η*_*b*_ = 10^–5^ and *η*_*f*_ = 10^–2^ for the backbone *θ* ^*b*^ and classifiers $\theta_{i}^{f}\;\forall i$ , respectively. Unless otherwise specified, all the algorithms employ a batch size equal to 32 for each group trained, mainly because of the hardware memory limitations. For the distributed version, we additionally adopted a warm-up scheme by doubling the learning rate for the first three iterations. We searched the optimal number of local steps using *J* ∈ {1, 10, 100} and found *J* = 10 to be the best; similarly, the slow momentum values *β* ∈ {0.1, 0.3, 0.5, 0.7} were evaluated before choosing *β* = 0.3. To provide meaningful comparisons, we considered a fixed wall-clock time budget of 60 h per experiment with reference to using NVIDIA GTX1080 GPUs.

#### 5.1.3 Testing procedure

To assess the performances of the algorithms, we selected the model that performed best on the SF-XL validation set and used it to measure the Recall@1 (R@1) and Recall@5 (R@5) values. Following standard procedures (Zaffar et al., 2021; Schubert et al., 2023), Recall@N is defined as the number of queries for which at least one of the first N predictions is correct, divided by the total number of queries. A prediction is deemed correct if its distance from the query is less than 25 m (Arandjelović et al., 2018). In reporting the final performance, we tested the chosen model on the Pitts250k (Torii et al., 2015), Pitts30k (Gronát et al., 2013), Tokyo 24/7 (Torii et al., 2018), Mapillary Street Level Sequences (MSLS) (Warburg et al., 2020), SF-XL (Berton et al., 2022a), St. Lucia (Milford and Wyeth, 2008), SVOX (Berton et al., 2021), and Nordland (Sünderhauf et al., 2013) datasets.

### 5.2 D-CosPlace vs CosPlace

In this section, we compare the results obtained by D-CosPlace with those from the original CosPlace algorithm in terms of both convergence speed (cf. Table 2) and final model quality given the time budget (cf. Table 1).

**TABLE 1:** Final model quality comparisons between CosPlace and D-CosPlace for equal training times on several VG datasets and varying numbers of CosGroups used during training. The results show that D-CosPlace can leverage multiple CosGroups, outperforming the vanilla CosPlace on average. The best overall results for each dataset are shown in boldface, while the best result for each number of CosGroups is underlined.

Method	#CosGroups	Pitts-30k		Pitts-250k		Tokyo 24/7		MSLS		SF-XL v1		SF-XL v2		Average	
		R@1	R@5	R@1	R@5	R@1	R@5	R@1	R@5	R@1	R@5	R@1	R@5	R@1	R@5
CosPlace (Berton et al., 2022a)	4	89.4	95.0	90.5	97.1	80.0	89.5	81.0	87.7	65.6	73.0	85.6	91.8	82.0	89.0
D-CosPlace		89.6	94.8	90.4	96.6	77.8	90.8	83.0	89.5	67.6	76.0	85.4	92.5	82.3	90.0
D-CosPlace (w/SlowMo)		90.0	95.0	90.6	96.6	**81.3**	**91.7**	82.2	89.5	68.5	75.5	85.4	92.3	83.0	90.1
CosPlace (Berton et al., 2022a)	8	89.5	94.8	90.4	96.9	81.6	90.2	81.8	88.7	65.5	74.1	84.6	91.6	82.2	89.4
D-CosPlace		90.1	**95.2**	91.4	**97.3**	80.3	89.8	83.2	89.9	70.4	78.8	86.4	93.6	83.6	90.8
D-CosPlace (w/SlowMo)		90.0	**95.2**	**91.5**	96.9	80.9	**91.7**	83.3	89.8	70.4	78.9	86.6	94.0	83.8	91.1
CosPlace (Berton et al., 2022a)	16	89.4	94.9	90.4	96.7	78.4	89.2	81.5	88.2	64.5	73.4	84.8	91.5	81.5	89.0
D-CosPlace		**90.3**	**95.2**	91.1	96.9	80.6	89.5	83.0	89.9	69.2	78.9	86.6	93.3	83.5	90.6
D-CosPlace (w/SlowMo)		90.0	95.0	91.3	97.2	78.4	90.8	**84.1**	**90.4**	**71.2**	**79.7**	**88.1**	**94.2**	**86.4**	**91.2**

#### 5.2.1 Convergence speed

We compared the convergence speed of D-CosPlace to that of the vanilla CosPlace. For both algorithms, we report the wall-clock training times under the same conditions using a single GPU and 4 GPUs separately. The results in Table 2 show that D-CosPlace achieves the same final accuracy as that of CosPlace while requiring less than half of the time budget. This is because the proposed parallel training procedure avoids training instabilities due to changing the CosGroup, thus leveraging the potential of the classification proxy task in a more efficient manner.

**TABLE 2:** Convergence speed comparisons between CosPlace and D-CosPlace using $\bar{G}={\left\{G_{i}\right\}}_{i=1}^{8}$ (best results in boldface): D-CosPlace can achieve the same accuracy as CosPlace for a fraction of the total wall-clock time. Alternatively, it surpasses the performance of the vanilla CosPlace within the time budget.

Method	Wall-clock time (hh:mm)		Best accuracy (SF-XL val)	
	Target R@1	Best R@1	R@1	R@5
CosPlace (Berton et al., 2022a)	57:30	57:30	90.9	95.5
D-CosPlace J = 1	42:00	**49:50**	91.4	96.2
D-CosPlace J = 10	**25:50**	59:25	**92.2**	**96.6**
D-CosPlace J = 100	26:02	54:26	91.6	96.5

#### 5.2.2 Final model quality

In addition to being significantly faster, D-CosPlace also achieves a better final model quality within the time budget. Table 1 shows that the distributed version consistently outperforms the vanilla baseline on all the tested datasets. The reason behind this rather prominent gap is that our formulation effectively implements the objective function in Eq. 2 while CosPlace implements Eq. 2.

#### 5.2.3 Scalability on the number of CosGroups

To further corroborate the claim that our formulation of CosPlace training is effective for exploiting larger datasets, we present the results for various numbers of training groups. It is noted that the original CosPlace treats $\bar{G}$ as a hyperparameter and determined that $\bar{G}={\left\{G_{i}\right\}}_{i=1}^{8}$ worked best, whereas adding more groups would be detrimental. The results in Table 1 confirm this limitation of CosPlace and show that D-CosPlace can effectively utilize more CosGroups, owing to the formulation of the objective function of Eq. 2.

#### 5.2.4 Fair comparison with larger batch size

**FIGURE 4:**
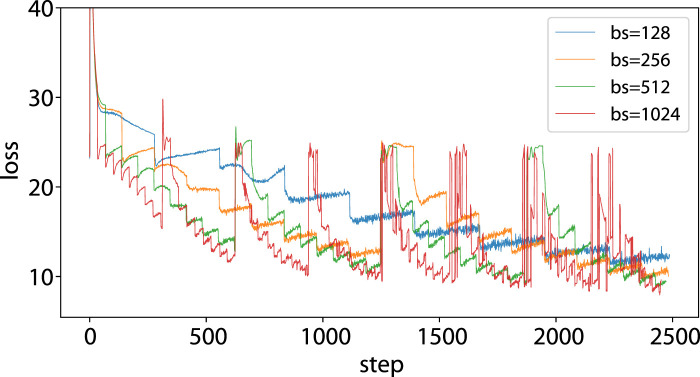
Noisy loss during training of a CosGroup in CosPlace: the training loss is plotted for the first 2.5*k* steps, which correspond to an iteration with batch size 128 and two iterations with batch size 256. It can be observed that the stepwise behavior remains even after enlarging the batch size, suggesting that other factors may be involved. The abrupt jumps observed for the orange, green, and red lines are attributed to the changes in the trained CosGroups (and hence the final classification head), which occur in fewer steps with respect to the blue line, owing to the increase in batch size.

Since the distributed version trains *N*_*t*_ groups in parallel using the same original batch size for all groups (e.g., respective classifiers), the actual batch size with respect to *θ* ^*b*^ is *N*_*t*_ times larger than that used for the vanilla CosPlace. For fair comparison, we also implemented CosPlace with the same batch size to investigate if a larger batch size would be needed to achieve faster convergence. The results presented in Figure 4 show that there is no advantage in increasing the batch size for the convergence speed or final model quality, further corroborating that CosPlace’s problem lies in the sequential training procedure.

### 5.3 Ablation study: effect of local steps

Local steps ensure that the distributed training is more efficient from a communication perspective by lowering the synchronization frequency. However, even when a large number of local steps is desirable, too many steps could slow the convergence when the training distributions are different, like in our case. For this reason, *J* is treated as a hyperparameter. Table 2 shows the impact of the local steps on the convergence speed and final model quality, where the former is expressed in terms of wall-clock time to reach the accuracy of the vanilla CosPlace and the latter is expressed as R@1/R@5. It can be seen that *J* = 10 produces the optimal balance between training time, convergence speed, and final model quality.

### 5.4 Comparisons with other methods

#### 5.4.1 Baselines

Herein, we compare D-CosPlace with a number of SOTA VPR methods, namely, the evergreen NetVLAD Arandjelović et al. (2018), SFRS Ge et al. (2020) that improves on NetVLAD with an ingenious augmentation technique, Conv-AP Ali-bey et al. (2022) that uses a multisimilarity loss Wang et al. (2019), CosPlace Berton et al. (2022a), and MixVPR Ali-bey et al. (2023) that uses a powerful and efficient MLP-mixer as the aggregator. For NetVLAD and SFRS, we use the authors’ best-performing backbone, which is the VGG16 (Simonyan and Zisserman, 2015), whereas for all the other methods, we use their respective implementations with a ResNet-50 backbone and output dimensionality of 512.

#### 5.4.2 Results

As seen from the results in Table 3, D-CosPlace not only improves upon the vanilla CosPlace by a large margin of +11.5% on average R@1 but also achieves new results as a SOTA VPR algorithm, surpassing CONV-AP by +1.6% on average R@1. These results show that the improved formulation of the classification proxy task originally introduced in CosPlace effectively learns better features for image retrieval.

**TABLE 3:** Final model quality comparisons with state-of-the-art VPR approaches on several datasets using ResNet-50 as the backbone. The best overall results for each dataset are in boldface, and the second-best results are underlined. D-CosPlace outperform the competitors (including CosPlace) in all cases except the “Tokyo 24/7″ and “MSLS” datasets. We believe that this may be attributed to the superior fitting capabilities of D-CosPlace as well as the datasets being particularly different from the one used to train the models. However, D-CosPlace outperforms CosPlace by a large margin (+11.5% on R@1) on average.

Method	Pitts30k		Pitts250k		Tokyo 24/7		MSLS		SF-XL v1		SF-XL v2		St. Lucia	
	R@1	R@5	R@1	R@5	R@1	R@5	R@1	R@5	R@1	R@5	R@1	R@5	R@1	R@5
NetVLAD (Arandjelović et al., 2018)	85.0	92.1	85.9	93.1	69.8	81.3	58.9	70.8	40.0	52.9	76.9	88.8	64.6	80.3
SFRS (Ge et al., 2020)	89.1	94.6	90.4	96.3	80.3	88.6	70.0	80.0	50.3	60.0	83.8	90.5	75.9	86.6
Conv-AP (Ali-bey et al., 2022)	89.1	94.6	90.4	96.7	61.3	77.8	82.3	90.3	41.8	53.1	64.0	81.2	99.1	99.99
CosPlace (Berton et al., 2022a)	90.2	95.2	91.7	97.0	**89.5**	**94.9**	**86.9**	**93.2**	76.7	82.5	89.0	95.3	99.2	99.99
MixVPR (Ali-bey et al., 2023)	90.4	95.4	**93.0**	**97.8**	78.4	86.7	83.6	91.5	57.7	70.3	84.3	91.6	99.2	99.99
**D-CosPlace (proposed)**	**91.2**	**95.7**	92.3	97.3	85.7	94.0	86.1	91.9	**80.9**	**86.2**	**91.0**	**95.7**	**99.5**	**100.0**
	Nordland		SVOX night		SVOX overcast		SVOX rain		SVOX snow		SVOX sun		Average	
NetVLAD (Arandjelović et al., 2018)	13.1	21.1	8.0	17.4	66.4	81.5	51.5	69.3	54.4	71.8	35.4	52.7	58.5	67.2
SFRS (Ge et al., 2020)	16.0	24.1	28.6	40.6	81.1	88.4	69.7	81.5	76.0	86.1	54.8	68.3	66.6	75.8
Conv-AP (Ali-bey et al., 2022)	66.5	79.7	51.6	68.8	90.0	96.6	87.3	94.7	89.5	97.0	75.9	88.3	83.4	91.0
CosPlace (Berton et al., 2022a)	59.2	74.6	36.0	52.5	90.5	95.9	80.3	90.0	86.4	95.3	75.3	88.1	73.5	83.3
MixVPR (Ali-bey et al., 2023)	**67.2**	**81.0**	44.8	63.2	93.9	97.7	86.4	93.9	**93.9**	97.6	78.7	91.2	80.9	89.7
**D-CosPlace (proposed)**	65.6	79.7	**56.5**	**73.0**	**94.6**	**97.8**	**88.8**	**96.1**	91.1	**97.8**	**81.9**	**91.9**	**85.0**	**92.1**

### 5.5 Open challenges

Our analysis in Section 3.3 reveals that CosPlace’s training procedure experiences severe jumps in the loss function due to the optimization procedure not implementing the objective function in Eq. 2 correctly. Indeed, the sharp jumps in loss occur only in the vanilla CosPlace because of the training process that optimizes different CosGroups (and their related classification heads) one at a time. This does not occur in D-CosPlace since all classifiers associated with the CosGroup are jointly optimized (Figure 3). A second challenge that we experienced with CosPlace is the noisy optimization of a single CosGroup, as shown by the loss in Figure 4. It is noted that the training loss is particularly unstable and remains high for many steps before dropping abruptly, with a seemingly periodic cycle every ≈1k steps. We initially associated this behavior with the batch size, especially if it is a low value when compared to the output dimensionality of the final layer. Each CosGroup is in fact associated with ≈35k classes on average, which makes the problem hard to learn. Additionally, the LMCL loss seeks a hard margin boundary, which can be difficult to achieve in high-dimensional problems. To validate this hypothesis, we increased the batch size to fill the memory of an NVIDIA-V100-32 GB GPU. The results in Figure 4 show that the problem persists even after increasing to 1,024 samples. Considering the validation results, the initial value of 32 still gives the best validation performance, substantiating the conclusion that increasing the batch size is not a practical solution. This difficulty of learning a single CosGroup is still present in D-CosPlace since the optimization with respect to a CosGroup is the same as that for CosPlace. We believe this to be an intrinsic limitation of the classification approach of CosPlace that will be an interesting direction for future works.

## 6 Conclusion

In this work, we analyzed the training procedure of CosPlace, a recent SOTA large-scale VPR method, by showing that its sequential protocol does not correctly implement the intended objective. By leveraging an incremental perspective on the problem, we modified the training procedure such that it correctly optimizes the learning objective function. This new formulation enables efficient distributed training since it allows disjoint sets of the dataset to be preallocated to the assigned compute nodes and benefits from the multiple local training steps. In particular, we show that i) D-CosPlace converges faster than CosPlace and that ii) within a fixed time budget, D-CosPlace outperforms CosPlace by a large margin. We also outline some open challenges in further speeding up the training of CosPlace, highlighting the instabilities during the training of the CosGroups. We believe that these insights are valuable for the research community in not only the field of VPR but also other large-scale image retrieval tasks.

## Data Availability

The original contributions presented in the study are included in the article/Supplementary material; further inquiries can be directed to the corresponding author.
